# Prorenin receptor acts as a potential molecular target for pancreatic ductal adenocarcinoma diagnosis

**DOI:** 10.18632/oncotarget.10583

**Published:** 2016-07-13

**Authors:** Arivajiagane Arundhathi, Wen-Han Chuang, Jen-Kun Chen, Shin-E Wang, Yi-Ming Shyr, Jiun-Yu Chen, Wei-Neng Liao, Hsin-Wei Chen, Yi-Min Teng, Chiao-Chih Pai, Chih-Hong Wang

**Affiliations:** ^1^ Department of Biological Science and Technology, National Chiao Tung University, Hsinchu, Taiwan; ^2^ Institute of Biomedical Engineering & Nanomedicine, National Health Research Institutes, Miaoli, Taiwan; ^3^ General Surgery, Taipei Veterans General Hospital, and National Yang Ming University, Taipei, Taiwan

**Keywords:** prorenin receptor, pancreatic cancer, PanIN lesions, Kras, SPECT/CT

## Abstract

Recent studies have implicated the prorenin receptor (PRR) is associated with pancreatic tumorigenesis. We therefore investigated the role of PRR in pancreatic tumorigenesis and assessed whether PRR can serve as a target for imaging diagnosis at early stages of PDAC. Here we show that aberrant expression of PRR in premalignant PanIN lesions, and human PDAC samples, and PDAC cell lines, particularly in Panc-1 cells. Interestingly, PRR expression was positively associated with PDAC progression. Moreover, overexpression of human PRR resulted in increased cell proliferation and decreased apoptosis, while knockdown of human PRR caused decreased cell proliferation and enhanced apoptosis in pancreatic cancer cells. We also observed that overexpression of human PRR enhanced MAPK and PI3K/Akt signaling pathways in PDAC cells, while knockdown of human PRR suppressed both of pathways. The confocal imaging analysis showed that human PRR was highly expressed in Panc-1, ASPC, and Miapaca cells, whereas BXPC-3, and HPAC cells had a significantly lower fluorescent signals. Consistently, the single-photon emission computed tomography (SPET/CT) showed that the uptake of anti-PRR labelled with ^125^I was higher in Panc-1 and ASPC tumors-bearing mice after 96 hours injection. Importantly, tumors in pancreas of *Pdx1-cre; LSL-Kras^G12D^* mice had a significant increased PRR expression and accumulation of radioactivity at 96 h after injection. These data suggest that ^125^I-anti-PRR can detect the orthotopic tumors in *Pdx1-cre; LSL-Kras^G12D^* mice. Therefore, anti-PRR labelled with ^125^I is a promising radiotracer for imaging diagnosis at early stages of pancreatic cancer.

## INTRODUCTION

Pancreatic ductal adenocarcinoma (PDAC) is the fourth leading cause of cancer death in the United States and the median overall 5-year survival rate is less than 5% due to the aggressive nature of pancreatic cancer [1–3]. Most PDAC patients are caused by Kras mutation, and are diagnosed at an advanced stage, and only 10% of patients are resectable at the time of presentation [4]. Although the molecular mechanism of PDAC has well known in past decade, it is extremely difficult to diagnose PDAC at an early stages and reduce mortality of pancreatic cancer [5]. Therefore, discovery of novel targets for PDAC to aid early diagnosis are urgently needed.

The (pro)renin receptor (PRR), a transmembrane protein, named ATP6ap2, is located on the X chromosome in human, and encodes a 350-amino acid protein with a single transmembrane domain [6]. PRR is a new component of renin angiotensin system (RAS) and has high affinity with prorenin and renin resulting in cleavage of angiotensinogen and signaling through the mitogen-activated protein kinase (MAPK) and Wnt/β-catenin signaling pathways [7]. Moreover, PRR is mainly expressed in kidney, heart, pancreas, brain, blood vessels, macrophages, T cells and granulocytes [8–10]. Although several studies have shown that PRR is involved in renal and heart pathophysiology such as diabetic nephropathy, kidney ischemic, cardiac fibrosis [11, 12], the role in pancreas remains unclear. More recently, PRR plays an important role in the pathogenesis of PDAC development [13]. The PRR is highly expressed in the metastasis of pancreatic cancer through activation of the Wnt/β-catenin signaling pathway, while PRR knockdown by siRNA triggers apoptosis of PDAC, and causes decreased cell proliferation [13]. However, it is still unknown whether PRR is a potential molecular target for imaging diagnosis at early stages of PDAC.

Single-photon emission computed tomography (SPET/CT), a noninvasive molecular imaging technique using radiolabeled compounds, is a useful tool for carcinogenic processes commonly diagnosis and staging of human tumors [14]. SPET/CT has been considered being more accurate in the detection of the primary and metastasis tumors [15]. Administration of a radiolabelled antibody to the cancer patients has been applied for diagnosis the tumors at early stages [16]. Therefore, the aims of this study are to determine whether PRR is able to detect human PDAC at early stages, and iodine-125 (^125^I)-labeled PRR can serve as a novel target for human PDAC.

In this study, we developed a noninvasive *in vivo* imaging technique to measure tumor PRR expression. We used anti-PRR was radiolabeled with ^125^I, and tumor targeting was analyzed by SPECT/CT in mice bearing subcutaneous human pancreatic cancer xenografts, and genetically engineered mouse model of pancreatic cancer, *Pdx1-cre; LSL-Kras^G12D^* mice. We have generated the *Pdx1-cre; LSL-Kras* mouse strain by breeding LSL-Kras^G12D^ “floxed” mice, with *Pdx-Cre* transgenic mice, which express Cre recombinase from pancreas-specific Pdx1 promote [17]. Our results show that anti-PRR labelled with ^125^I (^125^I-anti-PRR) was significantly accumulated in tumor site of xenografts mice and *Pdx1-cre; LSL-Kras^G12D^* mice. Thus, our findings suggest that ^125^I-anti-PRR can serve as a potential target for imaging diagnosis of human PDAC.

## RESULTS

### PRR is highly expressed in human pancreatic intraepithelial neoplasia (PanIN) lesions, PDAC, and human pancreatic cancer cell lines

**Figure 1:**
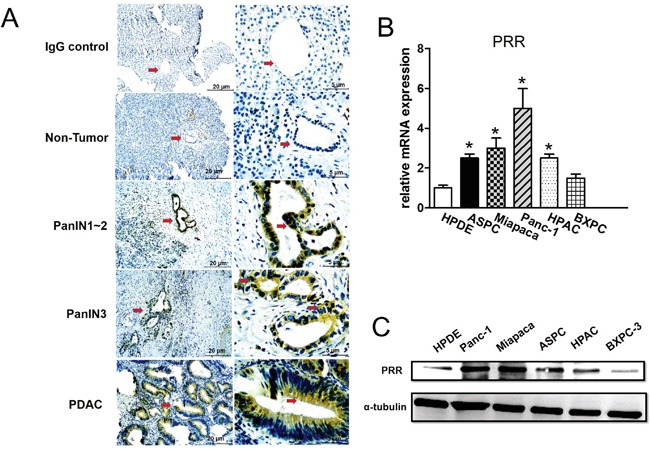
Expression of Prorenin Receptor (PRR) by Immunohistochemical analysis, qPCR and western blot **A.** Immunohistochemical (IHC) analyses of human PRR in PDAC samples from patients. The PRR was highly expressed in human PanIN3 and pancreatic cancer, but not in normal pancreatic cells. **B.** mRNA expression of human PRR analyzed by qPCR in HPDE, and pancreatic cancer cells (ASPC, Miapaca-2, Panc-1, HPAC and BXPC-3). **C.** Western blot analysis of PRR in pancreatic cancer cell lines (HPDE, ASPC, Miapaca-2, Panc-1, HPAC and BXPC-3). Data are means ± SEM. *, p<0.05 vs. HPDE cells.

Immunohistochemical (IHC) analysis showed that PRR is significantly expressed in the ductal of neoplastic epithelial cells in 90 samples of human PDAC tissues (Figure 1A). We observed that human PDAC tissues had the highest levels of PRR expression, while non-tumor tissues had the lowest levels of PRR expression (Figure 1A). In addition, we also found that PRR was overexpressed in PanIN1-2 and PanIN-3 lesions where atypical nuclei were observed (Figure 1A). Therefore, the data suggest that aberrant PRR expression may occur at early stages of pancreatic tumorigenesis. Interestingly, we also investigated the PRR expression in pancreatic cancer cell lines. The five human pancreatic cancer cell lines (Panc-1, ASPC, BXPC-3, HPAC, and MIAPaCa-2) and human normal pancreatic ductal epithelial cells (HPDE) were analyzed by real-time PCR (qPCR) and western-blot. We found that the mRNA levels of *PRR* were significantly increased in the five human pancreatic cancer cell lines (Panc-1, ASPC, BXPC-3, HPAC, and MIAPaCa-2) than that of HPDE cells (Figure 1B). These data was consistent with protein levels of PRR that was higher in Panc-1, MIAPaCa-2, HPAC, BXPC-3, and ASPC than that of HPDE (Figure 1C). Therefore, the findings suggest that pancreatic cancer may result in increased PRR expression.

### Enhanced PRR expression in PDAC patients

**Figure 2:**
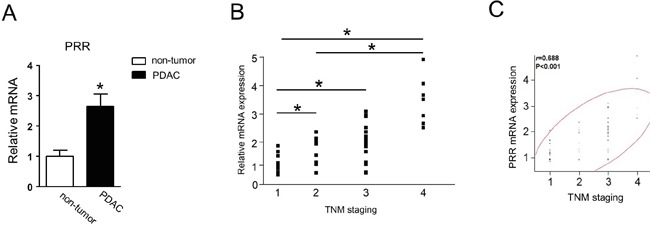
Human PRR is strongly expressed in PDAC and correlates with TNM staging **A.** Human PRR was 2.5-fold expressed in PDAC (n=90). **B.** human PRR expression was along with TNM staging (n=90). **C.** human PRR expression was positively associated with TNM staging. Data are means ± SEM. *, p<0.05 vs. non-tumor cells.

Next, we studied the 90 samples from PDAC patients. We found that PRR gene expression was significantly increased 2.5-fold in PDAC tissues compared to non-tumor tissues (Figure 2A). We noted that PRR expression was increased along with TNM staging (Figure 2B). After multivariate analysis, we found that PRR expression was associated with TNM staging and its correlation was 0.688 (P< 0.001) (Figure 2C). This data indicate that PRR expression is positively associated with TNM staging of human PDAC.

### Overexpression and downregulation of PRR affects cell proliferation and apoptosis in human PDAC

**Figure 3:**
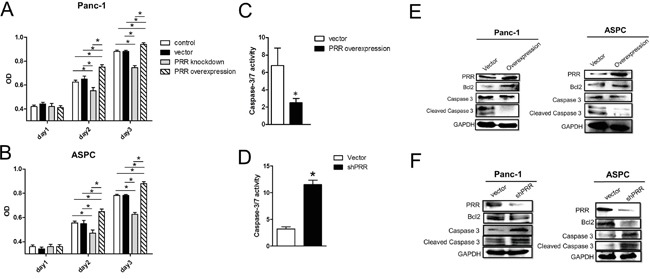
Effects of overexpression and downregulation of PRR on Panc-1 and ASCP cells in cell proliferation and apoptosis **A** and **B.** Cell proliferation was analyzed by MTT assay in overexpression or knockdown of human PRR in Panc-1 and ASCP cells compared to control vector (n=6). Results shown are shown the mean growth ± SEM. **C** and **D.** caspase 3/7 activity was investigated in in overexpression or knockdown of human PRR in Panc-1 and ASCP cells compared to control vector (n=5). **E** and **F.** Apoptosis biomarkers, Bcl2, caspase 3 and cleaved caspase 3, was measured by western blot analysis in overexpression or knockdown of human PRR in Panc-1 and ASCP cells compared to control vector. Data are means ± SEM. *, p<0.05 vs. control vector.

To explore role of PRR in cell proliferation and apoptosis, we generated overexpression and downregulation of human PRR gene in both of Panc-1 and ASPC cells. We cultured 3×10^3^ cells for MTT assay and found that overexpression of human PRR gene in both of Panc-1 and ASPC caused increased cell proliferation, while knockdown of human PRR in both of Panc-1 and ASPC resulted in decreased cell proliferation at day 2 and day3 (Figure 3A and 3B). Consistently, the caspase 3/7 activity was decreased in overexpression of human PRR in Panc-1 cells, while knockdown of human PRR in Panc-1 cells resulted in increased caspase 3/7 activity (Figure 3C and 3D). Western blot analysis also revealed that the overexpression of human PRR in both Panc-1 and ASPC cells caused reduced protein levels of cleaved caspase 3 and increased B-cell lymphoma 2 (Bcl2), while knockdown of human PRR in both of Panc-1 and ASPC cells resulted in increased protein levels of cleaved caspase 3 and decreased Bcl2 (Figure 3E and 3F). These data suggest that PRR may play a key role in cell proliferation and apoptosis in human PDAC.

**Figure 4:**
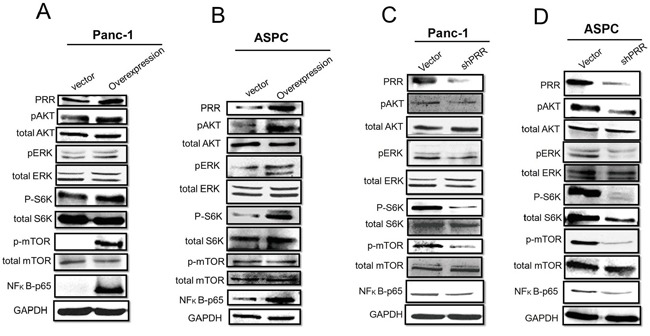
Role of PRR in MAPK and PI3K/Akt pathways Western results have shown in **A.** overexpression of human PRR in Panc-1 cells, **B.** overexpression of human PRR in ASPC, **C.** knockdown of human PRR in Panc-1cells, and **D.** knockdown of human PRR in ASPC cells.

Furthermore, several studies have shown that PRR-mediated angiotensin II-independent ROS formation is associated with activation of the MAPK/ERK1/2 and PI3/Akt signaling pathways [18]. We observed that overexpression of human PRR in both Panc-1 and ASPC cells caused enhanced phosphorylation of Akt, Erk1/2, ribosomal protein S6 kinase (S6K), mammalian target of rapamycin (mTOR) as well as increased NF-kB levels, while knockdown of human PRR in both of Panc-1 and ASPC cells resulted in decreased (Figure 4). These data indicate that PRR may be through MAPK and PI3K/AKT pathways to work its function.

### Analysis of PRR expression in human PDAC using confocal microscopy and SPECT/CT

**Figure 5:**
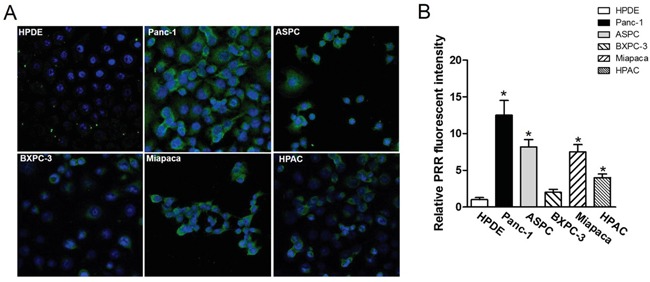
Confocal fluorescence microscopy imaging of PRR in pancreatic cancer cell lines Panc-1, ASPC, BXPC-3, Miapaca-2, and HPAC as well as normal pancreas HPDE **A.** human PRR expression in pancreatic cancer cell lines: Panc-1, ASPC, BXPC-3, Miapaca-2, and HPAC as well as normal pancreas HPDE using confocal fluorescence microscopy. **B.** Quantitation of fluorescence intensity by image J. All images were acquired under the same condition and displayed at the same scale. Magnification: 60x. Data are means ± SEM. *, p<0.05 vs. HPDE cells.

**Figure 6:**
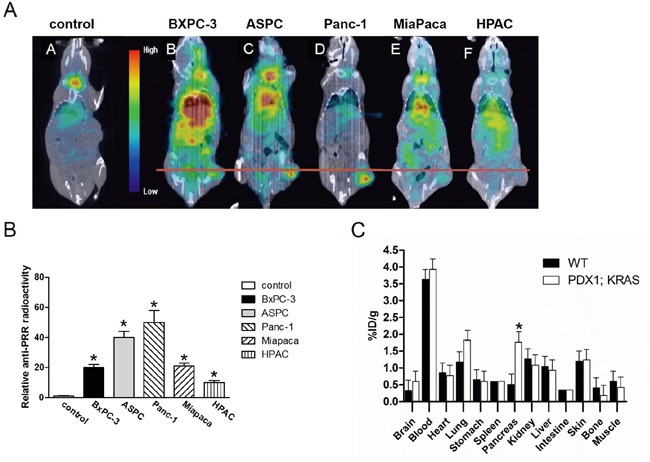
Antibody binding ability of PRR using SPECT/CT imaging of mice bearing pancreatic tumor xenografts The ^125^I-anti-PRR was applied for anti-PRR binding ability to tumors. **A.** The SPECT/CT imaging in wild-type (control), BXPC-3, ASPC, Panc-1, Miapaca-2, and HPAC. **B.** Quantitation of radioactivity intensity by image J. All images were acquired under the same condition and displayed at the same scale. **C.** Biodistribution of PRR labelled with ^125^I isotope in *Pdx1-cre; LSL-Kras^G12D^* mice (n=4). Data are means ± SEM. *, p<0.05 vs. wild-type mice.

We used the FITC-conjugated anti-PRR IgG1 antibody and DAPI to do confocal microscopic analysis in five pancreatic cancer cell lines and HPDE cells. We found that BXPC-3 and HPAC cells had lower PRR expression (green color), while Panc-1, ASPC, and Miapaca cells had higher PRR expression (Figure 5). Consistently, we also found the same results from SPECT/CT analysis (Figure 6A and 6B). We used anti-PRR labelled with ^125^I radioisotope (^125^I-anti-PRR). The nude mice were injected with Panc-1, ASPC, BXPC-3, Miapaca, and HPAC cells (1×10^6^ cells) with matrigel (1:1) into right flank for 6 weeks. The uptake of ^125^I-anti-PRR was higher in Panc-1, ASPC, and Miapaca tumor-bearing mice than in BXPC-3 and HPAC after 96 hours injection (Figure 6A and 6B). The SPECT/CT images were accurately demonstrated the uptake of ^125^I-anti-PRR in the tumor site. Importantly, the ^125^I-anti-PRR can discriminate to PRR expression from low to high in HPAC, BXPC-3, Miapaca, ASPC, and Panc-1 *in vivo*. Our results suggest that ^125^I-anti-PRR has highly specific binding ability to tumor site when pancreatic cancer cells express PRR.

### Biodistribution Studies

**Figure 7:**
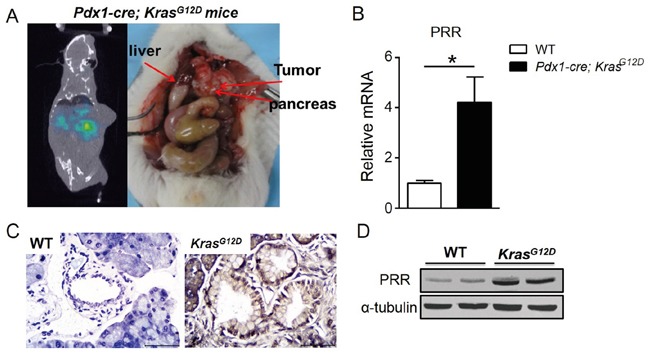
PRR expression in *Pdx1-cre; LSL-Kras^G12D^* mice **A.** The SPECT/CT imaging of *Pdx1-cre; LSL-Kras^G12D^* mice after 96-hr injected with 5 mCi (18.5 MBq) of ^125^I-anti-PRR through tail vein. **B.** PRR mRNA levels in *Pdx1-cre; LSL-Kras^G12D^* and wild-type mice. **C.** Immunohistochemical analyses of human PRR in pancreas of *Pdx1-cre; LSL-Kras^G12D^* and wild-type mice. **D.** Protein levels of PRR in *Pdx1-cre; LSL-Kras^G12D^* and wild-type mice.

We also used human pancreatic cancer mouse model, *Pdx1-cre; LSL-Kras^G12D^* mice for biodistrubution study. We have generated the *Pdx1-cre; LSL-Kras^G12D^* mouse strain by breeding LSL-Kras^G12D^ “floxed” mice, with Pdx1-Cre transgenic mice, which express Cre recombinase from pancreas-specific Pdx1 promoter [19]. All mice (wild-type and *Pdx1-cre; LSL-Kras^G12D^*; *n=6*) were analyzed by qPCR, IHC, and western-blot. We found that mRNA and protein levels of PRR were significantly increased in pancreas of *Pdx1-cre; LSL-Kras^G12D^* mice compared to wild-type mice (Figure 7B, 7C, and 7D). The mice were intravenous injected with ^125^I-anti-PRR through tail vein. Besides the tumors in pancreas (~2 %ID/g), the blood (~4 %ID/g), and lung (~2 %ID/g) also had a significant accumulation of radioactivity at 96 h after injection, as expected for a radiolabeled antibody that typically has a long circulation half-life and hepatic clearance (Figure 6C). Importantly, tumors in pancreas of *Pdx1-cre; LSL-Kras^G12D^* mice had a significant accumulation of radioactivity at 96 h after injection (Figure 7A). These data suggest that ^125^I-anti-PRR was able to detect the orthotropic tumors in *Pdx1-cre; LSL-Kras^G12D^* mice and PRR antibody labelled with ^125^I is a promising radiotracer for imaging diagnosis at early stages in human pancreatic cancer.

## DISCUSSION

The present study revealed PRR acts as a potential molecular target for PDAC diagnosis. To our knowledge, this is the first demonstration that PRR might be as a target for SPECT/CT imaging of pancreatic cancer. Several studies have shown that PRR plays an important role in tumorigenesis including pancreatic cancer [13]. In human pancreatic cancer samples analysis, we observed that PRR was highly expressed at early stages of human PanIN lesions, and human PDAC, whereas it was not expressed in normal pancreatic ductal cells. We also found that PRR was significantly expressed in human pancreatic cell lines, ASPC, Miapaca-2, Panc-1, HPAC and BxPC-3 compared with normal pancreatic cells HPDE. Through SPECT/CT imaging, Panc-1, ASPC, Miapaca, HPAC, and BXPC-3 cells xenografted to nude mice by ^125^I-labelled anti-PRR, and displayed a clear and specific accumulation of radioactivity in mice harboring subcutaneous Panc-1, ASPC, or Miapaca pancreatic tumors xenografts after 96 h. Importantly, the biodistribution study using human pancreatic cancer mouse model with engineered mutant *Kras^G12D^* activation revealed the uptake of ^125^I-anti-PRR was markedly elevated in pancreas of *Pdx1-cre; LSL-Kras^G12D^* mice than WT control littermates at 4 months of age. Thus, our findings indicate that elevated PRR is able to monitor for pancreatic tumor growth and may serve as a promising target for pancreatic cancer diagnosis.

PanIN is a histologically well-defined precursor to invasive PDAC, associated with active Kras mutations [20]. Previously study has demonstrated that PRR expression was barely detectable in normal pancreatic ducts, but aberrant expression of PRR was found in PDAC [13]. In the present study, the immunohistochemical staining showed the early stages of pancreatic carcinogenesis PanIN-1, PanIN-2 and PanIN-3 lesions had a significant increase of PRR expression, while PDAC had the highest expression levels of PRR, suggesting that inappropriate augmentation of PRR is essential for the survival and proliferation in early stages of pancreatic carcinogenesis. Consistently, the human pancreatic cancer cell lines ASPC, Miapaca-2, Panc-1, HPAC and BxPC-3 had highly expressed PRR compared with normal pancreatic cells HPDE. Importantly we found that PRR was strongly correlated with PDAC TNM staging and PRR expression was along with TNM staging. These results indicate PRR expression is associated with PDAC progression, and its expression was correlated with PDAC progression. However, the molecular mechanism for PRR regulation in PDAC progression remains unclear. Several studies have shown that PRR-mediated angiotensin II-independent ROS formation is associated with activation of the MAPK/ERK1/2 and PI3/Akt signaling pathways [18]. Moreover, PRR is a newly discovered component of the renin-angiotensin system (RAS) which has associated with diabetic nephropathy, hypertension and insulin resistance. Hypertension is caused by increased RAS activation together with upregulation of PRR via PI3K-Akt activation of cAMP response element-binding protein 1, and NF-κB p65 transcription factors. Therefore, knockdown of PRR may cause decreased levels of ERK1/2, AKT and NF-κB p65. Indeed, we found that PRR knockdown causes decreased levels of ERK1/2, AKT and NF-κB p65 in pancreatic cancer cells (Panc-1 and ASPC cells).

Moreover, our results support the hypothesis that PRR was significantly expressed in the early stages of PDAC and PRR knockdown triggered apoptosis resulting in decrease of the proliferative ability of human PDAC cells. Studies in tissue-specific PRR knock-out mice showed that specific deletion of PRR in cardiomyocytes [21] or glomerular podocytes [22] resulted in death a few weeks after birth. Downregulation of PRR led to apoptosis of cancer cells, indicating that PRR plays an important role in tumor survival. Indeed, PRR knockdown by shRNA caused significantly decreased pancreatic cancer cell proliferation and increased caspase 3/7 activity. Thus, PRR plays an important role in the pathogenesis of PDAC development.

Pancreatic cancer is often diagnosed in the very late stage, which is associated with its 5-year survival rate of about 5%, and a median survival of less than 6 months [23]. Because of the lack of specific symptoms and limitations in diagnostic methods, pancreatic cancer is nearly undetectable during its formative stages [24]. Pancreatic cancer cells are naturally resistant to current chemotherapy and radiation therapy, and to date, most known pancreatic cancer antigens have generated a relatively weak immune response. Early detection methods are under development but do not yet exist for pancreatic cancer. The correlation between PRR and PDAC progression makes PRR a useful marker for imaging diagnosis at early stages of pancreatic cancer. Therefore, the development of a SPECT/CT tracer for PRR imaging is of critical importance.

In the present study, we have successfully developed a PRR targeted for SPECT/CT imaging of pancreatic cancer. We first confirmed that PRR antibody binding ability by laser scan confocal microscopy imaging. The *in vitro* cell immunofluorescence experiment showed strong evidence of specific PRR antibody expression was significantly increased in Panc-1, and ASPC cells. Consistently, *in vivo* we radioiodinated and evaluated a PRR antibody in mice harboring subcutaneous Panc-1, ASPC, Miapaca, HPAC, and BXPC-3 pancreatic tumors xenografts using SPECT/CT and found that Panc-1 and ASPC tumors uptake and retention of radioactivity with the ^125^I-anti-PRR had significantly increased than others. Importantly, PRR is a membrane receptor protein that is able to activate intracellular signaling in tumorigenesis [25, 26]. The biodistribution study using human pancreatic cancer mouse model harboring *Kras^G12D^*, which causes activating mutation of the *Kras* oncogene and is the most frequent and the earliest genetic alteration associated with human pancreatic cancer [27]. The data revealed the uptake of ^125^I-anti-PRR was markedly accumulated in pancreas of *Pdx1-cre; LSL-Kras^G12D^* mice than in WT control littermates despite high signals in blood and lung tissue. There are several possible explanations by which accumulation of antibody in circulating. First, it is possible that the PRR antibody was not enough time for binding to tumor area resulting in PRR antibody exists in blood and lung tissue. Second, in most organs with a high number of blood vessels provide a continuous supply of oxygen, nutrients, and growth factors, while pancreas has a lower number of blood vessels that may affect antibody to target in tumor site. Third, cancer-associated stroma may restrain circulating antibody to target tumor site in *Pdx1-cre; LSL-Kras^G12D^* mice because cancer-associated stromal cells have been reported to cause lower FDG uptake in higher vascular perfusion [28]. However, we believe that a direct labeling with PRR antibody could allow us for targeted imaging in pancreatic cancer and PRR is a potential molecular target for PDAC diagnosis.

In conclusion, we have shown that PRR was significantly expressed in human PDAC. PRR expression was strongly associated with TNM staging, and PRR expression was along with PDAC progression. Moreover, the ^125^I-anti-PRR exhibited excellent cell uptake, and retention in Panc-1 and ASPC tumors xenografts using SPECT/CT. The biodistribution study demonstrated excellent tumor-targeting efficacy and specificity of PRR antibdoy in *Pdx1; Kras^G12D^* mouse model. Thus, the ^125^I-anti-PRR is a promising radiotracer for imaging diagnosis at early stages of pancreatic cancer.

## MATERIALS AND METHODS

### Human tissue samples

The institutional committee for ethics of National Chiao Tung University approved our studies using human tissue samples and informed consent was obtained from all subjects before the study was started. Eligible subjects were defined in advance as 90 patients with PDAC who were admitted to the Taipei Veterans General Hospital, Taipei, Taiwan, between July 2013 and August 2014. The protocol was performed in accordance with Declaration of Helsinki principles.

### Generation of *Pdx1-cre; LSL-Kras^G12D^* mice

The 4-week old of LSL(lox-STOP-lox) Kras^G12D^ mice on C57BL/6 background crossed with Pdx1-cre mice to generate the *Pdx1-cre; LSL-Kras^G12D^* mice. The Kras^G12D^ mice are a G12D mutation that is introduced in exon 1 of the mice *Kras* allele and contains a STOP cassette flanked by loxP sites (lox-STOP-lox ; LSL) which can be removed by cre-recombinase to allow the expression of the mutated *Kras* allele. Pdx1-cre mice express cre-recombinase in pancreas under the control of pancreas specific pdx1 promoter. To generated the *Pdx1-cre; LSL-Kras^G12D^* mice, we used the *LSL-Kras^G12D^* mice crossed with *Pdx1-cre* transgenic mice. Both *LSL-Kras^G12D^* and *Pdx1-cre* mice were purchased from Jackson laboratory. The experimental protocols of this study were reviewed and approved by the Institutional Animal Care and Use Committee (IACUC) of NHRI (approval number: NHRI-IACUC-102082-A).

### Cell culture and conditioned medium

Pan-1, BXPC-3, ASPC, HPAC, and MIAPaCa-2 cells were obtained from the Food Industry Research and Development Institute (FIRDI, Hsinchu, Taiwan). Panc-1, was grown in DMEM (Dulbecco's modified Eagle's medium) medium with 10% fetal bovine serum, penicillin (50 U/mL) and streptomycin (50 μg/mL, GIBCO, Carlsbad, CA, USA). BxPC-3, ASPC, HPAC, and MIAPaCa-2 were grown in RPMI-1640 medium (GIBCO, Carlsbad, CA, USA) supplemented with 10% fetal bovine serum (GIBCO, Carlsbad, CA, USA), penicillin (50 U/mL) and streptomycin (50 μg/mL, GIBCO, Carlsbad, CA, USA). HPDE cells were cultured in Keratinocyte-SFM medium (Life Technologies, Carlsbad, CA, USA).

### Quantitative reverse transcriptase polymerase chain reaction

For Quantitative reverse transcription polymerase chain reaction (qRT-PCR), total RNA isolation and reverse transcription were performed. Oligonucleotides were designed using Primer 3 software. Primers for human PRR were 5′- CATTGTCCATGGGCTTCTCT-3′ (sense), and 5′- GCATTCTCCAAAGGGTACGA-3′ (antisense), and mouse PRR were 5′-TTTGGATGAACTTGGGAAGC-3′ (sense), and 5′-CACAAGGGATGTGTCGAATG-3′ (antisense).

### Construction of overexpressing PRR gene in Panc-1 and ASPC cells

A pcDNA3.1-EGFP vector (Clontech Laboratories Inc., Mountain View, CA) was used in the construction of human PRR. In brief, the human PRR coding sequence was polymerase chain reaction (PCR)-amplified and PCR fragment was cloned into pGEM-T Easy vector (Promega, Sydney, NSW, Australia). The 1.05-kb BamHI- and SpeI-digested insert was then subcloned into the pcDNA3.1-EGFP expression vector to generate pEGFP-human PRR, which encodes enhanced green fluorescent protein (EGFP) and human PRR. 20 μg of pEGFP-human PRR plasmid was introduced into 5×10^6^ Panc-1 or ASPC cells using Lipofectamine 2000 (Invitrogen), and then pancreatic cancer cells were recovered in complete medium overnight and then cultured in the presence of 400 μg/ml of G418. Clones resistant to G418 selection were expanded, and expression levels were assessed by either fluorescence microscopy or quantitative fluorescence-activated cell sorting (FACS) analysis. To obtain stably transfected Panc-1 or ASPC cells that express similar levels of GFP, FACS was performed to isolate GFP-positive cells. Cells expressing GFP were excited at 488 nm and detected using a 530 nm.

### Transient gene transfection by siRNA and plasmid DNA

For short hairpin RNA (shRNA) PRR constructs, the sequence of shRNA was 5′-CCGGGGAACGAGTTTAGTATATTAACTCGAGTTAATATACTAAACTCGTTCCTTTTTG-3′. The lentiviral packaging and transduction shRNA PRR-endoding lentiviral vectors were constructed and packaged as described [29]. In short, 293T cells were transfected with pLSLPw-shRNA constructs along with packaging plasmids, pVSVG (addgene) and pLV-CMV-delta 8.2 (addgene) using Lipofectamine 2000 (Invitrogen). Virus-containing supernatants were collected at 48 to 96 h post-transfection and used to infect Panc-1 cells in the presence of 5 mg/ml polybrene (Sigma). After 24 h, the virus-containing medium was replaced with selection medium containing 5 mg/ml puromycin (Sigma). After cell growth was stable, the cells were used in the experiments.

### Western blot analysis

Total protein extracts (30 μg) were electrophoretically separated using 10% SDS-polyacrylamide gels and transferred onto nitrocellulose membranes. The gel contains acrylamide, Tris-HCl, 0.1% SDS, 0.1% APS, and TEMED. Then electrophoretically transferred proteins to polyvinylidene fluoride (PVDF) membranes (Bio-Rad Laboratories, Inc., Hercules, California, USA). Non-specific binding sides were blocking by 5% skim milk or BSA in TBST at 4°C overnight. Primary antibody added into the blocking buffer and incubated at 4°C overnight. Non-specific binding antibody washed away by TBST for 5 minutes 3 times. Secondary antibody which conjugated with HRP targeted the primary antibody PRR (GeneTex International Corporation, Hsinchu City, Taiwan), Bcl2 (Cell Signaling), caspase 3 (Cell Signaling), phosph-Akt (Cell Signaling), total Akt (Cell Signaling), phosph-mTOR (GeneTex International Corporation, Hsinchu City, Taiwan), total mTOR (GeneTex International Corporation, Hsinchu City, Taiwan), phosph-ERK (GeneTex International Corporation, Hsinchu City, Taiwan), total ERK (GeneTex International Corporation, Hsinchu City, Taiwan), p65 (Cell Signaling), GAPDH (GeneTex International Corporation, Hsinchu City, Taiwan), alpha-tubulin (GeneTex International Corporation, Hsinchu City, Taiwan) at room temperature for 1 hour. After 3 times washed, added the ECL (enhanced chemiluminescence) (Bio-Rad Laboratories, Inc., Hercules, California, USA) and quantified by imagination system.

### *In vivo* tumorigenicity studies

Mice were anesthetized with isoflurane. Panc-1, BXPC-3, ASPC, HPAC, and MIAPaCa-2 cells, 1 × 10^5^ (expressing scrambled shRNA or PRR shRNA), in 200 μL of PBS were injected subcutaneously into the upper right flanks using a 26G needle (n = 8 per group). Size of local tumors at the implanting site was measured with an electric caliper. Volumes of tumors were calculated as follows: tumor volume (mm^3^) = length × (width)^2^ × 0.5.

### MTT assays for PDAC cell counting to determine cell proliferative ability

A 200 μL aliquot of cells (3 × 10^3^ cells/mL in 10% FBS and 1% P+S containing media) was added to a 96 well plate and incubated for 24 hours at 37 °C in a humidified incubator containing 5% CO_2_ in air. After incubation with 2 μL renin(1 nM, 10 nM and ddH_2_O for control) for 24 hours, the medium was aspirated and a 20 μL MTT solution (PanReac AppliChem, Darmstadt, Germany, 5 mg/mL in PBS) was added to each well and the incubation continued for 4 hours. After this time the solution in each well was carefully removed. The purple formazan crystalline precipitate in each well was dissolved in 200 μL of Dimethyl sulfoxide (DMSO, Amresco, Solon, Ohio, USA). The visible absorbance of each well was quantified at 560 nm using a microplate photometer. Cell proliferation used optical density (OD) at 560 nm at day 1 to day 3.

### Immunohistochemistry of PRR

Immunohistochemistry of PRR was performed using surgical human specimens of PDAC. Briefly, paraffin sections were deparaffinized and incubated with methanol containing 0.3% hydrogen peroxide for 15 min, then 10% normal goat serum (Jackson ImmunoResearch Inc., West Grove, PA, USA) was added to the sections to block non-specific staining. The sections were incubated with an anti-human PRR rabbit polyclonal antibody (1:400 dilution, GeneTex International Corporation, Hsinchu City, Taiwan) for 1 h at room temperature. After washing with PBS, sections were also incubated with the secondary antibody (goat anti-rabbit IgG conjugated with horseradish peroxidase, (Jackson ImmunoResearch Inc., West Grove, PA, USA) for 40 min at room temperature. Sections were visualized by immersion in DAB (3,3 diaminobenzidine, Jackson ImmunoResearch Inc., West Grove, PA, USA) as a chromogen. Then nuclear staining was performed using hematoxylin, and each section was embedded.

### Detection of caspase-3/7 activity

Enzymatic activity of caspase-3/7 was measured using the Caspase-Glo 3/7 Assay kit (Promega, Madison, USA) according to the manufacturer's instruction. Briefly, pancreatic cells with or without overexpressing PRR or knockdown PRR were seeded in 96-well plate. Afterwards, the cells were lysed and incubated with 100 μL of Apo-ONE Caspase-3/7 reagent (substrate and buffer in the ratio of 1:100). After 1 h incubation in the dark at RT, the fluorescence of each well was measured at 485–520 nm by reading in an Epoch microplate reader (Biotek Instruments; Winooski, VT, USA). The experiments were performed in triplicate.

### Imaging probe preparation

The antibody of prorenin receptor (PRR) labelled with radioactive iodine (Na^125^I, PerkinElmer Inc., Waltham, MA) was performed by the precoated iodination tube (Thermo Fisher Scientific Inc., Rockford, lL). The antibody was purchased from GeneTex International Corporation, Hsinchu City, Taiwan. Tris iodination buffer (1 mL, containing 25 mM Tris-HCl, pH 7.5, 0.4 M NaCl) was added to prewet tube and decant at 1 min later. Then, Na^125^I (3.12 mCi) was added into precoated iodination tube and incubated for activation at room temperature for 6 min. The activated solution was mixed with 40 μg anti-PRR in 100 μL of Tris-buffer for 6 minutes at room temperature. To quench free ^125^I, 50 μL of tryrosine (10 mg/mL in Tris-buffer) as the scavenging buffer was added to the above-mentioned solution, which was mixed and incubated for 5 minutes with additional flicking at 1st and 4th minutes. The mixture was purified though Vivaspin® 500 ultracentrifugation device (100 kDa molecular weight cut-off, GE Healthcare UK Limited, Buckinghamshire, UK), which was washed twice using Tris-buffer and BSA solution (2.5 mg/mL). The concentrate was collected and diluted to 200 μL for injection. Radiochemical purity was surveyed by instant thin layer chromatography (ITLC) using chromatography paper (4 Chr, GE Healthcare UK Limited) as stationary phase and normal saline (0.9% NaCl) as mobile phase. Criteria of radiochemical purity is set as 95% for quality control.

### SPECT/CT imaging

CT and ^125^I-SPECT imaging were performed by a pre-clinical tri-modality imaging system (FLEX Triumph; Gamma Medica-Ideas, Northridge, CA, USA). The X-ray tube of CT was operated at voltage of 70 kVp, current of 0.185 mA, and geometrical magnification of 1.3. The 512 projections of CT data were reconstructed by filtered back projection (FBP) with a matrix of 512×512×512, presenting a pixel size of 180 μm. For SPECT imaging, 0.5 mCi (18.5 MBq) of ^125^I-antiPRR in 0.2 mL saline solution was injected into each mouse through tail vein. The SPECT images were taken at 2, 24, 48, and 96 h post-injection. Gamma photons were captured by dual head gamma camera fitted with high resolution parallel-hole collimators. Energy window was placed symmetrically around the 35.5 keV gamma photon peak of ^125^I. Gamma cameras were operated with 105 mm radius of rotation (ROR), 180-grade rotation, 64 projections and 12 s acquisition time per frame to collect data. SPECT images were reconstructed by filter back projection (FBP) using ramp filter (cutoff frequency=1) as a matrix of 80×80×64. SPECT image was registered to CT image and re-sliced as a matrix of 512×512×512.

### Biodistribution

Mice (wild-type and *Pdx1-cre; Kras^G12D^*) were anesthetized using isoflurane and subsequently intravenous injected with 5 μCi (0.185 MBq) of ^125^I-anti-PRR (4 μg) through tail vein. Mice were sacrificed at 96 hours. Blood and tissue samples including brain, heart, lung, stomach, spleen, pancreas, kidney, liver, intestine, skin, bone and muscle were collected for counting gamma rays of ^125^I by automatic gamma counter (2480 WIZARD2, PerkinElmer, Turku, Finland). Data of biodistribution were presented in %ID/g tissue wet weight. Experimental protocols of this study were reviewed and approved by the Institutional Animal Care and Use Committee (IACUC) of NHRI (approval number: NHRI-IACUC-102082-A).

### Statistical analysis

All values are expressed as mean ± SEM. Data were analyzed by Student's t test or analysis of variance (ANOVA). A multiple-comparison Tukey-Kramer post hoc test was performed with JMP software version 10 (SAS Institute Inc. NC, USA) to evaluate differences between groups. Multivariate linear regression analysis was performed to identify independent relationships. Before multivariate analyses were calculated, the distribution of the respective variables was tested for normality using a Kolmogorov - Smirnov test. A P value less than 0.05 was considered statistically significant.

## References

[1] Yachida S, Jones S, Bozic I, Antal T, Leary R, Fu B, Kamiyama M, Hruban RH, Eshleman JR, Nowak MA, Velculescu VE, Kinzler KW, Vogelstein B (2010). Distant metastasis occurs late during the genetic evolution of pancreatic cancer. Nature.

[2] Ghiorzo P (2014). Genetic predisposition to pancreatic cancer. World journal of gastroenterology.

[3] Whitcomb DC, Shelton C, Brand RE Genetics and Genetic Testing in Pancreatic Cancer. Gastroenterology.

[4] Li D, Xie K, Wolff R, Abbruzzese JL (2004). Pancreatic cancer. Lancet.

[5] Pettazzoni P, Viale A, Shah P, Carugo A, Ying H, Wang H, Genovese G, Seth S, Minelli R, Green T, Huang-Hobbs E, Corti D, Sanchez N (2015). Genetic events that limit the efficacy of MEK and RTK inhibitor therapies in a mouse model of KRAS-driven pancreatic cancer. Cancer research.

[6] Li Q, Raizada MK (2010). Is (pro)renin receptor a multifunctional receptor?. Hypertension.

[7] Bader M (2007). The second life of the (pro)renin receptor. Journal of the renin-angiotensin-aldosterone system.

[8] Geisberger S, Maschke U, Gebhardt M, Kleinewietfeld M, Manzel A, Linker RA, Chidgey A, Dechend R, Nguyen G, Daumke O, Muller DN, Wright MD, Binger KJ (2015). New role for the (pro)renin receptor in T-cell development. Blood.

[9] Wiwanitkit V (2011). (pro)renin receptor: A stable molecule. Journal of natural science, biology, and medicine.

[10] Ahmed BA, Seda O, Lavoie JL (2011). (Pro)renin receptor as a new drug target. Current pharmaceutical design.

[11] Oshima Y, Morimoto S, Ichihara A (2014). Roles of the (pro)renin receptor in the kidney. World journal of nephrology.

[12] Takahashi H, Ichihara A, Kaneshiro Y, Inomata K, Sakoda M, Takemitsu T, Nishiyama A, Itoh H (2007). Regression of nephropathy developed in diabetes by (Pro)renin receptor blockade. Journal of the American Society of Nephrology.

[13] Shibayama Y, Fujimori T, Nguyen G, Hirose T, Totsune K, Ichihara A, Kitada K, Nakano D, Kobori H, Kohno M, Masaki T, Suzuki Y, Yachida S (2015). (Pro)renin receptor is crucial for Wnt/beta-catenin-dependent genesis of pancreatic ductal adenocarcinoma. Scientific reports.

[14] Sharkey RM, Karacay H, McBride WJ, Rossi EA, Chang CH, Goldenberg DM (2007). Bispecific antibody pretargeting of radionuclides for immuno single-photon emission computed tomography and immuno positron emission tomography molecular imaging: an update. Clinical cancer research.

[15] Benard F, Turcotte E (2005). Imaging in breast cancer: Single-photon computed tomography and positron-emission tomography. Breast cancer research.

[16] Husarik DB, Steinert HC (2007). Single-photon emission computed tomography/computed tomographyfor sentinel node mapping in breast cancer. Seminars in nuclear medicine.

[17] Morton JP, Karim SA, Graham K, Timpson P, Jamieson N, Athineos D, Doyle B, McKay C, Heung MY, Oien KA, Frame MC, Evans TR, Sansom OJ (2010). Dasatinib inhibits the development of metastases in a mouse model of pancreatic ductal adenocarcinoma. Gastroenterology.

[18] Peng H, Li W, Seth DM, Nair AR, Francis J, Feng Y (2013). (Pro) renin receptor mediates both angiotensin II-dependent and -independent oxidative stress in neuronal cells. PloS one.

[19] Westphalen CB, Olive KP (2012). Genetically engineered mouse models of pancreatic cancer. Cancer journal.

[20] Grasso D, Garcia MN, Hamidi T, Cano C, Calvo E, Lomberk G, Urrutia R, Iovanna JL (2014). Genetic inactivation of the pancreatitis-inducible gene Nupr1 impairs PanIN formation by modulating Kras(G12D)-induced senescence. Cell death and differentiation.

[21] Kinouchi K, Ichihara A, Sano M, Sun-Wada GH, Wada Y, Kurauchi-Mito A, Bokuda K, Narita T, Oshima Y, Sakoda M, Tamai Y, Sato H, Fukuda K (2010). The (pro)renin receptor/ATP6AP2 is essential for vacuolar H+-ATPase assembly in murine cardiomyocytes. Circulation research.

[22] Ichihara A (2012). (Pro)renin receptor and autophagy in podocytes. Autophagy.

[23] Bagcchi S (2015). Urine test can detect early stage pancreatic cancer. The Lancet Oncology.

[24] Mayor S (2015). Immunotherapy improves overall survival in pancreatic cancer. The Lancet Oncology.

[25] Cruciat CM, Ohkawara B, Acebron SP, Karaulanov E, Reinhard C, Ingelfinger D, Boutros M, Niehrs C (2010). Requirement of prorenin receptor and vacuolar H+-ATPase-mediated acidification for Wnt signaling. Science.

[26] Glorioso N, Atlas SA, Laragh JH, Jewelewicz R, Sealey JE (1986). Prorenin in high concentrations in human ovarian follicular fluid. Science.

[27] Fendrich V, Schneider R, Maitra A, Jacobsen ID, Opfermann T, Bartsch DK (2011). Detection of precursor lesions of pancreatic adenocarcinoma in PET-CT in a genetically engineered mouse model of pancreatic cancer. Neoplasia.

[28] Alvarez R, Musteanu M, Garcia-Garcia E, Lopez-Casas PP, Megias D, Guerra C, Munoz M, Quijano Y, Cubillo A, Rodriguez-Pascual J, Plaza C, de Vicente E, Prados S (2013). Stromal disrupting effects of nab-paclitaxel in pancreatic cancer. British journal of cancer.

[29] Tseng PH, Matsuzawa A, Zhang W, Mino T, Vignali DA, Karin M (2010). Different modes of ubiquitination of the adaptor TRAF3 selectively activate the expression of type I interferons and proinflammatory cytokines. Nat Immunol.

