# Correction: Prorenin receptor acts as a potential molecular target for pancreatic ductal adenocarcinoma diagnosis

**DOI:** 10.18632/oncotarget.26597

**Published:** 2019-01-11

**Authors:** Arivajiagane Arundhathi, Wen-Han Chuang, Jen-Kun Chen, Shin-E Wang, Yi-Ming Shyr, Jiun-Yu Chen, Wei-Neng Liao, Hsin-Wei Chen, Yi-Min Teng, Chiao-Chih Pai, Chih-Hong Wang

**Affiliations:** ^1^ Department of Biological Science and Technology, National Chiao Tung University, Hsinchu, Taiwan; ^2^ Institute of Biomedical Engineering & Nanomedicine, National Health Research Institutes, Miaoli, Taiwan; ^3^ Department of General Surgery, Taipei Veterans General Hospital, and National Yang Ming University, Taipei, Taiwan; ^4^ Institute of Molecular Medicine and Bioengineering, National Chiao Tung University, Hsinchu, Taiwan

**This article has been corrected:** Due to an error in proofreading, the total ERK, AKT, and S6K in Figure 4A are incorrect. The proper Figure 4A is shown below. In addition, updates have been made to the 3rd affiliation and a 4th affiliation has been added. Both are shown below. The authors declare that these corrections do not change the results or conclusions of this paper.

^3^ Department of General Surgery, Taipei Veterans General Hospital, and National Yang Ming University, Taipei, Taiwan

^4^ Institute of Molecular Medicine and Bioengineering, National Chiao Tung University, Hsinchu, Taiwan

**Figure 4:**
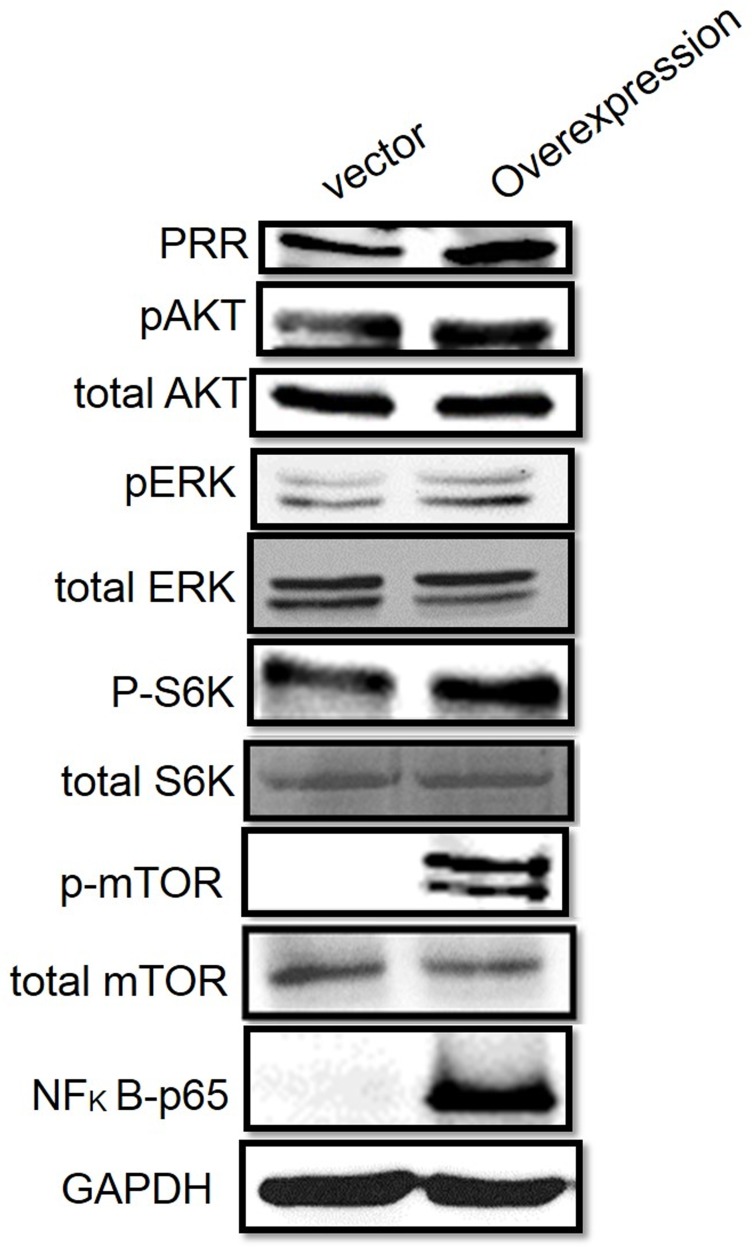
Role of PRR in MAPK and PI3K/Akt pathways Western results have shown in A. overexpression of human PRR in Panc-1 cells.

Original article: Oncotarget. 2016; 7:55437-55448. https://doi.org/10.18632/oncotarget.10583

